# Biosystem Analysis of the Hypoxia Inducible Domain Family Member 2A: Implications in Cancer Biology

**DOI:** 10.3390/genes11020206

**Published:** 2020-02-18

**Authors:** Celia Salazar, Osvaldo Yañez, Alvaro A. Elorza, Natalie Cortes, Olimpo García-Beltrán, William Tiznado, Lina María Ruiz

**Affiliations:** 1 Instituto de Ciencias Biomédicas, Facultad Ciencias de la Salud, Universidad Autónoma de Chile, Santiago 8910060, Chile; celia.salazar@uautonoma.cl; 2 Computational and Theoretical Chemistry Group, Departamento de Ciencias Químicas, Facultad de Ciencias Exactas, Universidad Andres Bello, Santiago 8370251, Chile; osvyanezosses@gmail.com (O.Y.); wtiznado@unab.cl (W.T.); 3 Institute of Biomedical Sciences, Faculty of Medicine and Faculty of Life Sciences, Universidad Andres Bello, Santiago 8370146, Chile; alvaro.elorza@unab.cl; 4 Millennium Institute on Immunology and Immunotherapy, Santiago 8331150, Chile; 5 Facultad de Ciencias Naturales y Matemáticas, Universidad de Ibagué, Carrera 22 calle 67, Ibagué 730002, Colombia; natalie.cortes@unibague.edu.co (N.C.); jose.garcia@unibague.edu.co (O.G.-B.)

**Keywords:** *HIGD2A*, cancer, DNA methylation, mRNA expression, miRNA, quercetin, hypoxia

## Abstract

The expression of *HIGD2A* is dependent on oxygen levels, glucose concentration, and cell cycle progression. This gene encodes for protein HIG2A, found in mitochondria and the nucleus, promoting cell survival in hypoxic conditions. The genomic location of *HIGD2A* is in chromosome 5q35.2, where several chromosomal abnormalities are related to numerous cancers. The analysis of high definition expression profiles of *HIGD2A* suggests a role for HIG2A in cancer biology. Accordingly, the research objective was to perform a molecular biosystem analysis of *HIGD2A* aiming to discover HIG2A implications in cancer biology. For this purpose, public databases such as SWISS-MODEL protein structure homology-modelling server, Catalogue of Somatic Mutations in Cancer (COSMIC), Gene Expression Omnibus (GEO), MethHC: a database of DNA methylation and gene expression in human cancer, and microRNA-target interactions database (miRTarBase) were accessed. We also evaluated, by using Real-Time Quantitative Reverse Transcription Polymerase Chain Reaction (qRT-PCR), the expression of *Higd2a* gene in healthy bone marrow-liver-spleen tissues of mice after quercetin (50 mg/kg) treatment. Thus, among the structural features of HIG2A protein that may participate in HIG2A translocation to the nucleus are an importin α-dependent nuclear localization signal (NLS), a motif of DNA binding residues and a probable SUMOylating residue. *HIGD2A* gene is not implicated in cancer via mutation. In addition, DNA methylation and mRNA expression of *HIGD2A* gene present significant alterations in several cancers; *HIGD2A* gene showed significant higher expression in Diffuse Large B-cell Lymphoma (DLBCL). Hypoxic tissues characterize the “bone marrow-liver-spleen” DLBCL type. The relative quantification, by using qRT-PCR, showed that *Higd2a* expression is higher in bone marrow than in the liver or spleen. In addition, it was observed that quercetin modulated the expression of *Higd2a* gene in mice. As an assembly factor of mitochondrial respirasomes, HIG2A might be unexpectedly involved in the change of cellular energetics happening in cancer. As a result, it is worth continuing to explore the role of *HIGD2A* in cancer biology.

## 1. Introduction

Mitochondria are crucial for virtually all aspects of malignant transformation and tumor progression, counting since the proliferation of transformed cells, the resistance of these cells to hostile environmental surroundings, the interaction of transformed cells with the tumor stroma, and their dissemination to remote anatomical sites [1]. Besides being the leading supplier of ATP, mitochondria could provide building blocks for the proliferation of malignant cells, they produce reactive oxygen species (ROS), and they are critical players in regulated cell death signaling [1].

Among the main mechanisms used by mitochondria for the malignant transformation of cells, first, there is the production of ROS, which favors the accumulation of potential oncogenic defects in DNA, and the activation of probable oncogenic signaling pathways [2]. Secondly, there is an abnormal accumulation of mitochondrial oncometabolite such as fumarate, succinate, and 2-hydroxyglutarate [3]. Thirdly, there are defects in the mitochondrial permeability transition (MPT), which allow the survival of malignant cells through the deregulation of regulated cell death processes [4]. Mitochondria influence the outcome of cancer cells to therapy through metabolic reprogramming between glycolysis and oxidative phosphorylation. The search for many anti-carcinogenic treatments is based on the identification of molecules that kill cancer cells or sensitize them to treatments by priming MPT [1]. 

Thus, the understanding of mitochondrial metabolism is fundamental in the development of new anti-cancer agents. Our research group is focused on the study of the Hypoxia Inducible Domain Family Member 2A, HIG2A, which is a small protein (106 amino acids) located in the inner membrane of the mitochondria. It has a hypoxia-induced-protein domain at the N-terminus [5]. HIG2A has a role in the respiratory supercomplexes assembly, a function that has been evidenced in the C2C12 mouse cell line, where the knockdown of *Higd2a* (nomenclature of mice gene) impaired supercomplex formation by the release of CIV [6,7]. Recently, we showed that the knockdown of *HIGD2A* (nomenclature of a human gene) decreases the activity of Complex I in the supercomplexes of HEK293 cells [8]. Noteworthy, in that study, the authors described the following results for the first time: the *Higd2a* gene exhibits differential expression in mice under basal physiological conditions that could be associated with different cell proliferation rates, and with differentiation and physiological oxygen levels in each tissue. Additionally, we also proved that physiological hypoxia induces *HIGD2A* (*Higd2a*) gene expression. Interestingly, the latter showed an increase during the cellular differentiation of C2C12 cells from myoblast to myotubes [8]. These results support a role for HIG2A in conditions of physiological stress, such as hypoxia in some tissues, and cell differentiation processes.

Further analysis of the *HIGD2A* gene promoter region in human chromosome 5 provided insights on how HIG2A could be related to cell cycle management. These studies evidenced several probable binding sites for different transcription factors related to cell cycle control, including E2F-1, E2F-2, E2F-3a, E2F-4, and E2F-5 [8]. These results agree with the evidence that under oxidative metabolism, E2F-1 directs cellular responses by acting as a regulatory switch from glycolytic to oxidative metabolism [9,10]. Moreover, we analyzed the effects of E2F-1 modulation on *HIGD2A* gene expression using roscovitine (inhibitor of CDKs), flavopiridol, and caffeic acid phenethyl ester (CAPE) (antiproliferative drugs) [8]. Roscovitine treatment significantly increased *HIGD2A* gene expression in the human embryonic kidney HEK293 cell line. Treatment with CAPE decreased *HIGD2A* gene expression in mouse myoblast C2C12 cells [8]. In the same work, the E2F-1 regulatory action in *HIGD2A* gene was studied, showing that the inhibition of cell proliferation treated with CAPE promotes E2F1 binding to the regulatory region of *HIGD2A*, thus setting a role for E2F-1 in the regulation of *HIGD2A* expression. Notably, analysis of *HIGD2A* genomic location showed a chromosome 5q35.2 section, a region where several chromosomal abnormalities are usually related to cancer [11,12,13,14]. 

Oncogenic mutations in the small GTPase Ras are highly prevalent in cancer. Depletion of *HIGD2A* selectively impairs the viability of colon adenocarcinoma cells (DLD1), which are Ras mutant cells, suggesting a role of HIG2A in cell cycle regulation and a potential target in cancer therapy [15]. Furthermore, the analysis of high definition expression profiles of *HIGD2A* with the Gene Expression Omnibus (GEO) repository [16,17] suggested a role for HIG2A in cancer biology. This analysis showed that *HIGD2A* expression is significantly increased in Methotrexate resistant colon cancer cell lines (HT29 resistant cells) (GDS3160) and Cisplatin-resistant non-small lung cancer cell lines (H460 resistant cells) (GDS5247). Additionally, when the estrogen receptor alpha is silenced in MCF7 breast cancer cells, a significant decrease of *HIGD2A* expression was evidenced (GDS4061). All the above data are suggesting a role of HIG2A in cell cycle regulation.

Accordingly, in light of the background mentioned above, the research objective was to perform a molecular biosystem analysis of *HIGD2A*, aiming to obtain insights on its implications in cancer biology.

## 2. Materials and Methods 

### 2.1. Datasets

The Gene Expression Omnibus (GEO) [16] repository for gene expression profiles of DLBCL was screened, and datasets were analyzed with GEO2R [17]. The microarray Illumina Human HT-12 V4.0 expression bead chips were used in the study; “Role of hypoxia in Diffuse Large B-cell Lymphoma: Metabolic repression and selective translation of HK2 facilitates development of DLBCL”. This study offers further conclusive proof of the contribution of HK2 in the development of B-cell lymphoma. It proposes that HK2 is a vital metabolic driver of DLBCL (Diffuse Large B-cell Lymphoma) phenotype. The authors contributed to the public dataset GSE104212 [18]. For this study, two human lymphoma cell lines, HLY-1 and SUDHL2, were cultured and assessed under hypoxic conditions (*n* = 3, biological replicates per cell line) or normoxia (*n* = 3, biological replicates per cell line), followed by a gene expression microarray analysis to examine the global gene expression differences under these conditions [18]. Another dataset analyzed was obtained with the Agilent-014850 Whole Human Genome Microarray 4 × 44K G4112F and were used in the study of gene-expression profiles in a series of non-Hodgkin lymphoma (NHL) patients (Dataset GSE32018). This study shows that PIM2 kinase inhibition is a logical process in DLBCL therapy and gives a new marker for patient stratification [19]. The gene-expression profiling from Dataset GSE32018 was conducted in a series of 114 B-cell non-Hodgkin lymphoma patients (DLBCL, Follicular Lymphoma (FL), Marginal Zone Lymphoma_Type (MALT), Mantle Cell Lymphoma (MCL), Chronic Lymphocytic Leukemia (CLL), and Nodal Marginal Zone Lymphoma (NMZL)). Seven freshly frozen lymph nodes and six freshly frozen reactive tonsils were used as controls [19]. The last Dataset GSE12453 obtained the expression profiling by array [HG-U133_Plus_2] Affymetrix Human Genome U133 Plus 2.0 Array was used in the study; origin and pathogenesis of lymphocyte-predominant Hodgkin lymphoma as revealed by global gene expression analysis. This study shows a relationship of microdissected lymphocytic and histiocytic (L&H) lymphoma cells to the origin from germinal center B cells at the transition to memory B cells. L&H cells are typified by abnormal ERK signaling and constitutive NF-κB activity [20]. The analysis of differential gene expression was performed in primary human lymphoma cells of Nodular Lymphocyte-Predominant Hodgkin Lymphoma (NLPHL) in comparison with primary lymphoma cells of classical Hodgkin lymphoma cells, and other B-non-Hodgkin Lymphoma (B-NHL) samples, and subsets of non-neoplastic B lymphocytes isolated from blood or tonsils [20].

### 2.2. In Silico Analysis

A homology modelling of HIG2A protein structure was generated with the SWISS-MODEL repository (https://swissmodel.expasy.org/repository/uniprot/Q9BW72?csm=205DE0AE39950053) [21,22]. Two crystal structures of backbone structure of human membrane protein HIGD1A and HIGD1B (protein data bank code: 2LON, 2LOM) were chosen as template for the construction of the three-dimensional HIG2A model (Model 1A and Model 1B). For validation, we used the PROCHECK program [23], which assesses the stereochemical quality of protein structures and the root mean square deviation (RMSD), superimposing the structures of proteins and calculating their deviation.

Additionally, we performed some in silico analysis for the prediction of nuclear localization signals (NLS) with the NLS Mapper software (nls-mapper.iab.keio.ac.jp) and DNA-binding residues in HIG2A protein with the DP-Bind software (lcg.rit.albany.edu/dp-bind), which is a web server for sequence-based prediction of DNA-binding residues in DNA-binding proteins. Moreover, we searched for post-translational modifications of HIG2A and with the GPS-SUMO prediction of SUMOylating sites and SUMOylating binding motifs (sumosp.biocuckoo.org).

DNA methylation and gene expression of *HIGD2A* in cancer was analyzed with MethHC, a database for human pan-cancer gene expression, methylation and microRNA expression [24] (http://methhc.mbc.nctu.edu.tw). The *HIGD2A* promoter was selected and the methylation level evaluation method was defined as maximum.

### 2.3. Immunofluorescence and Confocal Microscopy

The immunofluorescence analysis was performed according to the procedure previously reported [8] with brief addition for the inner nuclear membrane Lamin-B protein localization; anti-Lamin B antibody (Lamin B sc-6216 SANTA CRUZ BIOTECHNOLOGY, INC), the secondary antibody red signal-Alexa Fluor 546. Hoechst 33342 (Blue signal after DNA binding). Z-axis series were obtained using a Leica SP8 confocal microscopy.

### 2.4. Isolation of Mitochondria and Nucleus, and Western Blot

The isolation of mitochondria and nucleus and Western blot were performed according to the procedure previously reported [8].

### 2.5. Animals

The protocol of animal management was approved by the Bioethics Committee of the Vice-Rectory for Research and Postgraduate Studies of Universidad Andrés Bello, Approval Act 009/2010, of 8 July, 2010. The animals were treated and handled according to the Chilean National Commission for Scientific and Technological Research-CONICYT requirements for the care and use of laboratory, in accordance with NIH guidelines (The Guide for the Care and Use of Laboratory Animals, 1996). Male C57BL/6 mice were housed in groups of nine mice per cage and maintained at 22 °C on a 12:12-h light–dark cycle, with food and water ad libitum before the procedures. Moreover, male C57BL/6 mice (12 months of age) were daily injected intraperitoneally (i.p) with either 50 mg/kg quercetin (Sigma-Aldrich, Cat # Q4951, Merck KGaA, Darmstadt, Germany) (*n* = 9), or vehicle (5% DMSO) plus PBS (*n* = 9) for 15 days, according to the protocol previously described [25].

### 2.6. Reverse Transcription and Quantitative Real-Time PCR (qRT-PCR)

Total RNA was extracted from mice tissues with TRIzol^TM^ Reagent (Invitrogen, Thermo Scientific, Waltham, MA, USA) according to the manufacturer’s protocol. RNA quantification and quality assessment were determined using the spectrophotometer Infinite M200 Pro (TECAN AG, Zürich, Switzerland) and agarose electrophoresis. RNA (2 µg) was used for the reverse transcription with the RevertAid First Strand cDNA synthesis Kit (Thermo Scientific, Waltham, MA, USA). qPCR was performed using FastStart Essential DNA Green Master Kit (Roche, Risch-Rotkreuz, Zug, Switzerland) and the LightCycler^®^ 96-Real time PCR system (Roche, Risch-Rotkreuz, Zug, Switzerland). Data are presented as relative mRNA levels of *HIGD2A* normalized to *PPIA* mRNA levels. The primers used were: *HIGD2A* Fw: 5′-GCCTTTTGATCCGTCCAAGC-3′, Rev: 5′-CTGAAACGGAGGGAGCAAGT-3′; *PPIA* Fw: 5′-GTGGTCTTTGGGAAGGTG-3′, Rev: 5′-GGTGATCTTCTTGCTGGTC-3′. The thermal conditions used were as follows: an initial three-step amplification (95 °C for 10 s, 60 °C for 10 s and 72 °C for 10 s), followed by a one-step melting (95 °C for 10 s, 65 °C for 60 s and 97 °C for 1 s) and finishing with a one-step cooling (37 °C for 30 s). All reactions were concluded with an integrated melting curve reaction to verify the specificity of the amplification. Two experimental replicates were analyzed in a “LightCycler” run, improving the precision within the test. In order to improve the variation between assays, four runs were carried out on four different days (biological replicates).

### 2.7. Statistical Analysis

All statistical analyses were performed with the Graphpad Prisma 6 software (San Diego, CA, USA). An unpaired Student′s *t*-test followed by a Mann-Whitney test was used when comparing two average values. One way-ANOVA followed by a Dunnett´s multiple comparison test was also performed.

## 3. Results

### 3.1. Structural Features of HIG2A Protein 

**Figure 1:**
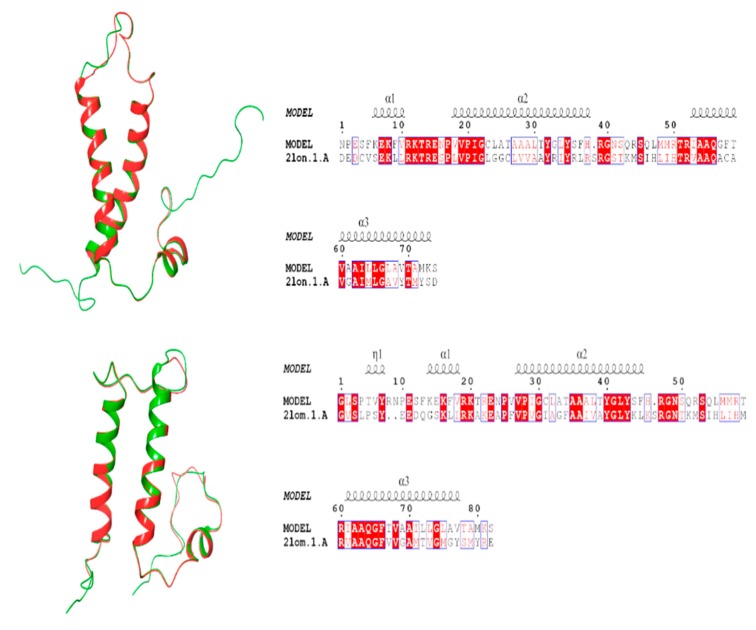
Images in the left side show superimposed template (green) on their respective model (red). Images in the right show alignment generated by the ESPript 3.0 webtool [27] of HIG2A protein with the PDBs: 2LON and 2LOM, accessions used to build the Models 1-A and 1-B.

**Figure 2:**
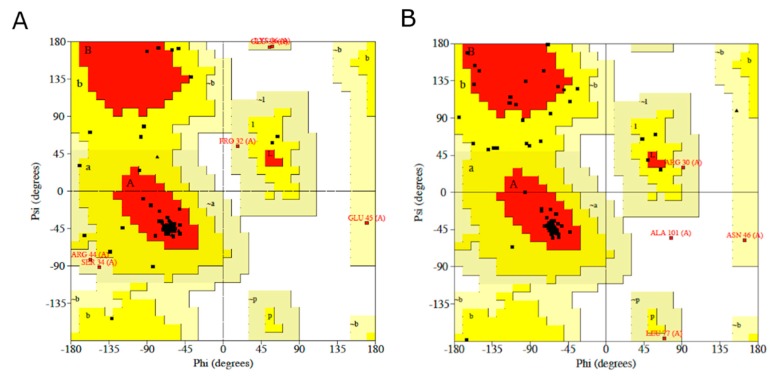
Ramachandran plots generated via PROCHECK for (**A**) HIG2A protein Model-1A and (**B**) HIG2A protein Model-1B. PROCHECK shows that the residues in most favored (red), additionally allowed (yellow), generously allowed (pale yellow) and disallowed regions (white color).

For the homology modeling of HIG2A protein, two crystal structures of backbone structure of human membrane protein HIGD1A and HIGD1B (protein data bank code: 2LON, 2LOM) were chosen as template for the construction of the three-dimensional HIG2A model (Model 1A and Model 1B) as it displayed a sequence identity of 36–36.14% and a similarity of 50.67–54.22%, see Figure 1. In the current study, the stereo-chemical evaluation of backbone psi and Phi dihedral angles of the HIG2A models showed that Model 1A and Model 1B residues were 70.3% and 70.4% in the most favorable region, and 0% and 14% in the additional allowed region, respectively (Table 1 and Figure 2). In general, a score close to 100% implies the good stereo-chemical quality of the model [26]. The total quality G-factor −0.29 and −0.23, for Model 1A and 1B, indicated a good quality model (acceptable values of the G-factor in PROCHECK are between 0 and −0.5, with the best models displaying values close to zero). The PROCHECK stereochemical analysis showed neither wrong contacts nor bad scores for main-chain or side-chain parameters. Therefore, these PROCHECK results suggest that the predicted model was of good quality.

**Figure 3:**
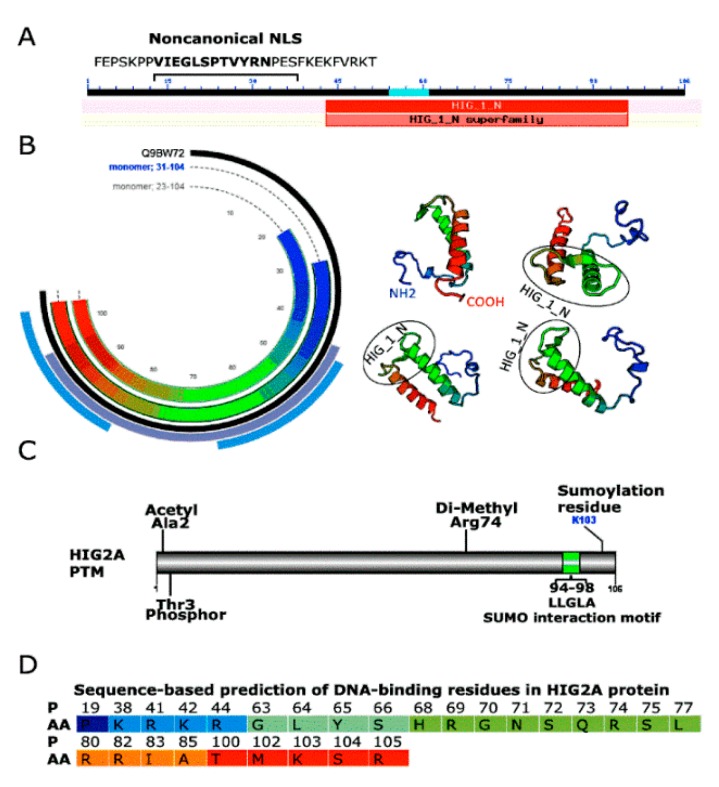
Structural features of HIG2A protein. (**A**) HIG2A noncanonical nuclear localization signals (NLS), (**B**) Q9BW72 (HIG2A_HUMAN) Homo sapiens (Human) from SWISS MODEL protein structure homology-modelling server, (**C**) post-translational modification of HIG2A, (**D**) sequence-based prediction of DNA-binding residues in HIG2A protein. P, position; AA, amino acid; No mitochondrial presequence; G 70 MPP cleavage site.

Our previous studies suggest that changes in oxygen concentration, cellular metabolism, and cell cycle regulate *HIGD2A* expression [8]. HIG2A protein might function as a regulator of respiratory supercomplexes assemblies in response to hypoxia, cellular metabolism, and cell cycle [8]. HIG2A could function as a hypoxia sensor in respiratory supercomplexes to activate signaling pathways of response to hypoxic stress. To explore the potential participation of HIG2A in cellular signaling pathways, we performed several analyses of the HIG2A protein sequence. With the nuclear localization signal, NLS Mapper software [28], for HIG2A, an importin α-dependent nuclear localization signal was predicted (Figure 3), which is a noncanonical NLSs recognized by importin α [29]. This NLS in HIG2A supports the participation of HIG2A in a cellular signaling pathway. HIG2A has a motif of DNA binding residues in the alpha-helix, which also supports the interaction of HIG2A with DNA (Figure 3).

**Figure 4:**
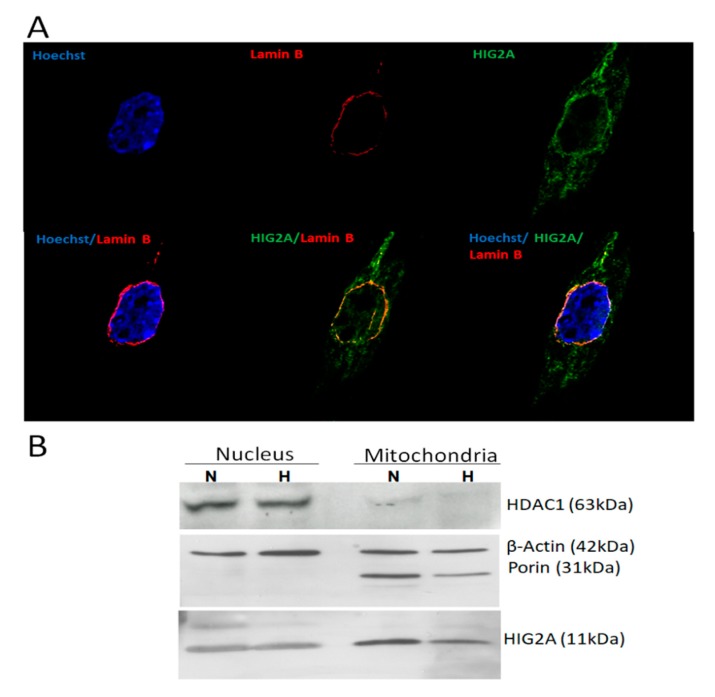
HIG2A protein localizes in mitochondria and nucleus. (**A**) Immunofluorescence image of an Anti-HIGD2A antibody stained C2C12 cells, the secondary antibody (Green signal, DyLight^®^ 488). Lamin B (sc-6216) stained showing nuclear lamina localization, the secondary antibody red signal-Alexa Fluor 546. Hoechst 33342 (Blue signal after DNA binding). z-axis series were obtained using a Leica SP8 confocal microscopy. (**B**) Western blot of HIG2A in mitochondria and nucleus protein extract of HEK293 cells. Normoxia condition (N), Hypoxia condition (H).

Moreover, we looked at post-translational modifications for HIG2A that account for their participation in signaling pathways. In high throughput, proteomic screening was found acetylation in Ala 2- [30], phosphorylation in Thr 3 [31], and di-methylation in Arg 74 (PhosphoSitePlus^®^) in HIG2A (Figure 3). HIG2A protein localizes in the mitochondrial network and nucleus [8]. The immunofluorescence analysis of C2C12 cells by confocal microscopy allows observing the colocalization of HIG2A with the inner nuclear membrane protein, Lamin-B (Figure 4A). With the Western blot, an upper band of approximately 10 kDa higher than HIG2A was detected in the nucleus fraction with Anti-HIG2A antibody, suggesting that this upper band could be a post-translational modification of HIG2A (Figure 4B). For this reason, protein HIG2A was analyzed for SUMOylating; a probable SUMO interaction motif and a SUMOylating nonconsensus residue were identified (Figure 3). The sumoylation could regulate the nuclear localization of some proteins [32,33,34,35].

### 3.2. Genetic Features of HIGD2A Gene in Cancer

**Figure 5:**
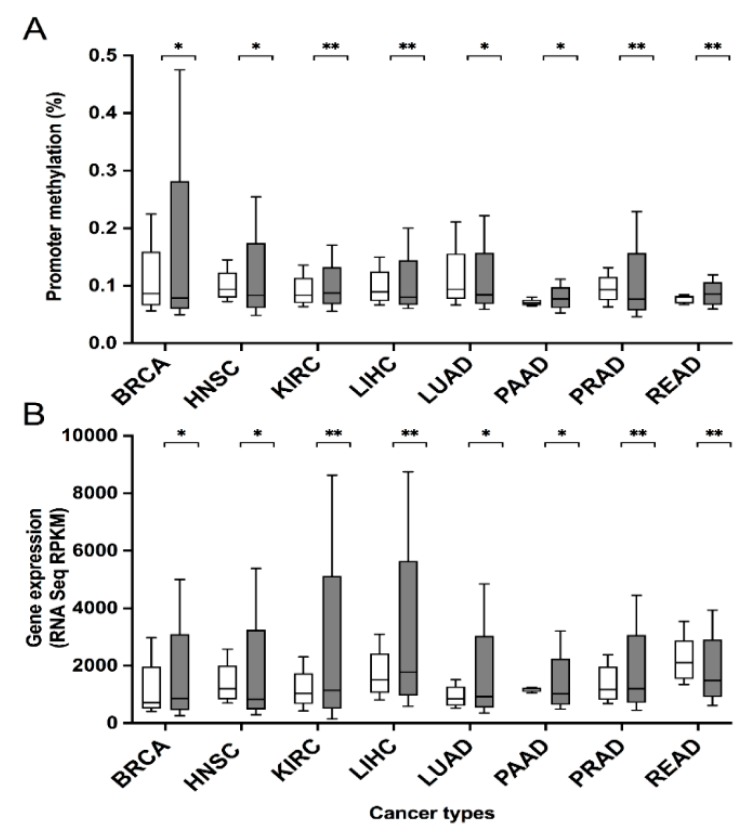
DNA Methylation (**A**) and mRNA Expression (**B**) in the *HIGD2A* gene in Cancer. The distinct methylation of *HIGD2A* in promoter region between cancer and normal tissues in GC patients (MethHC, a database of DNA methylation and gene expression in human cancer). The average beta value for the maximum methylation level evaluation method was used. Gene expression value was obtained from RNA Seq RPKM (Reads Per Kilobase per Million mapped reads) values in TCGA Data Portal by MethHC. Box plots in grey represent cancer samples and those in white represent normal samples. BRCA (*p*-value 0.020048563931465) cancer samples (*n* = 748), normal samples (*n* = 129); HNSC: Head and Neck Squamous Cell Carcinoma (*p*-value 0.0057123457160735), cancer samples (*n* = 517), normal samples (*n* = 67); KIRC: Kidney Renal Clear Cell Carcinoma (*p*-value 0.0010765698395251), cancer samples (*n* = 301), normal samples (*n* = 168); LIHC: Liver Hepatocellular Carcinoma (*p*-value 0.0014579663558734), cancer samples (*n* = 204), normal samples (*n* = 65); LUAD: Lung Adenocarcinoma (*p*-value 0.047325233745657), cancer samples (*n* = 452), normal samples (*n* = 48); PAAD: Pancreatic Adenocarcinoma (*p*-value 0.031511114364529), cancer samples (*n* = 91), normal samples (*n* = 16); PRAD: Prostate Adenocarcinoma (*p*-value 9.6625187628874E-7), cancer samples (*n* = 340), normal samples (*n* = 66); READ: Rectum Adenocarcinoma (*p*-value 0.002214301636065), cancer samples (*n* = 96), normal samples (*n* = 13). “∗” indicates being statistically significant with *p* < 0.05. “∗∗” indicates being statistically significant with *p* < 0.005.

The Catalogue of Somatic Mutations in Cancer (COSMIC) Cancer Gene Census (CGC) database indicates that the *HIGD2A* gene (COSG58129) has been reported as having mutations in 29 unique samples out of a total of 35183 samples; therefore, *HIGD2A* is not a known cancer-driving gene [36]. Moreover, mouse insertional mutagenesis experiments do not support the designation of *HIGD2A* as a cancer-causing gene [37]. On the other hand, DNA methylation is a vital epigenetic mechanism that stabilizes gene expression and cellular states; their alteration has a role in tumor initiation and evolution [38]. In the present study, we evaluated the correlation between DNA methylation and mRNA expression in the *HIGD2A* gene in cancer. For this purpose, we used the MethHC, a database of DNA methylation and gene expression in human cancer [24]. The comparison of average beta value in tumor samples, and matched normal samples, indicates significant alterations in DNA methylation and mRNA expression in the *HIGD2A* gene in diverse cancer: Breast Invasive Carcinoma (BRCA), Head and Neck Squamous Cell Carcinoma (HNSC), Kidney Renal Clear Cell Carcinoma (KIRC), Liver Hepatocellular Carcinoma (LIHC), Lung Adenocarcinoma (LUAD), Pancreatic Adenocarcinoma (PAAD), Prostate Adenocarcinoma (PRAD), and Rectum Adenocarcinoma (READ) (Figure 5). As shown in Figure 5, the correlation between DNA methylation and mRNA expression in the *HIGD2A* gene is significant for most of the cancers previously mentioned. The correlation and *p*-valued between DNA methylation and mRNA expression for each cancer are: BRCA (correlation: −0.023392995888958, *p*-value: 7.7715611723761E-16); HNSC (correlation: −0.038296830523789, *p*-value: 8.8817841970013E-16); KIRC (correlation: 0.026605097926286, *p*-value: 1.2061907028738E-9); LIHC (correlation: 0.03121030048201, *p*-value: 3.5347748061909E-9); LUAD (correlation: 0.00043286112981717, *p*-value: 2.246819973406E-7); PAAD (correlation: 0.058019469632158, *p*-value: 0.40190361732643); PRAD (correlation: −0.19807571454707, *p*-value: 0.0002748122809052); and READ (correlation: 0.21585872962767, *p*-value: 7.0291561460323E-8).

**Figure 6:**
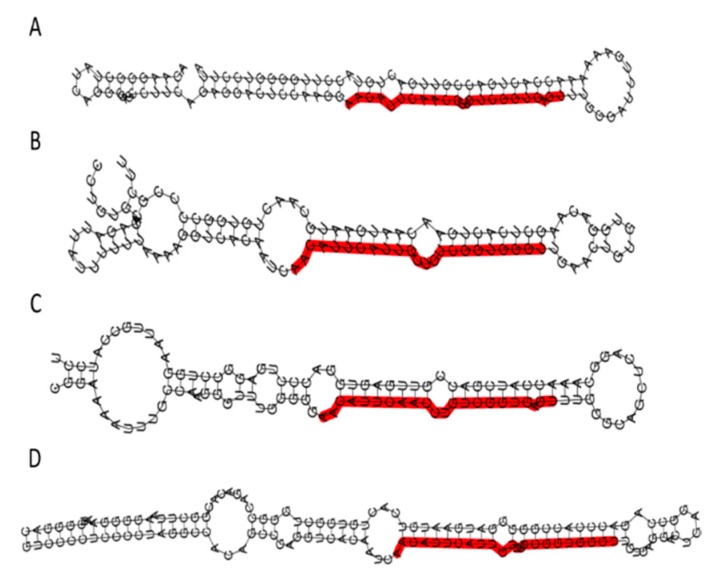
Secondary structure of pre-miRNA; hsa-mir-181a-2 (**A**), hsa-mir-181b-1 (**B**), hsa-mir-181c (**C**), and hsa-mir-181d (**D**) for *HIGD2A* target gene.

Moreover, we explored the microRNA-target interactions database (miRTarBase) analyzing microRNAs (miRNAs) for the *HIGD2A* gene [39]. These miRNAs are small non-coding RNAs that maintain cell homeostasis by negative regulation influencing each pathway practically from cell cycle checkpoint, cell proliferation to apoptosis [40]. Of the 17 miRNAs found, four miRNAs stand out as having experimental evidence and influence on different diseases related to cancer [39,41]. In Figure 6 the secondary structure of pre-miRNA; hsa-mir-181a-2, hsa-mir-181b-1, hsa-mir-181c, and hsa-mir-181d are presented. The word cloud of miRNA-disease information, for these miRNAs, are related to neoplasms, leukemia, carcinoma, lymphoma, among others. In Table 2, the significative clinical miRNA and gene expression profile (miRNA-Target expression profile) from The Cancer Genome Atlas (TCGA) is summarized. Briefly, the miRNA, hsa-mir-181a-2 prove a significant positive correlation for kidney chromophobe (KICH) and a negative correlation for PRAD. Besides, the miRNA, hsa-mir-181b-1, reveal a significant positive correlation for KICH and kidney renal papillary cell carcinoma (KIRP), and a negative correlation for HNSC, BRCA, and lung squamous cell carcinoma (LUSC). Moreover, the miRNA, hsa-mir-181c, indicates a significant positive correlation for KICH, liver hepatocellular carcinoma (LIHC), Cholangiocarcinoma (CHOL), and a negative correlation for BRCA and LUSC. Finally, the miRNA, hsa-mir-181d, demonstrate a significant positive correlation for LIHC and a negative correlation for BRCA and LUSC (Table 2).

### 3.3. Study of the Datasets of HIGD2A Expression in Diffuse Large B-cell Lymphoma by Profiling Arrays with Gene Expression Omnibus

Diffuse large B-cell lymphoma (DLBCL) is hematologic cancer and accounts for 35% to 40% of non-Hodgkin’s lymphomas, the most common malignant lymphoid disease in adults [42,43]. Several classification schemes have been proposed for DLBCL, one of which was the molecular profiling of DLBCL revealing three subtypes: mitochondrial oxidative phosphorylation (OXPHOS), B-cell receptor/proliferation, and host response [44]. Another more widely accepted classification scheme was the cell-of-origin (COO), which presented two categories based on patterns of gene expression reminiscent of germinal center B cell (GCB group) and activated B cell (ABC group) [45]. However, the different subtypes of DLBCL are associated with different pathogenic mechanisms and outcomes [43]. OXPHOS-DLBCLs shows increased glutathione levels, enhanced mitochondrial energy transduction, and greater incorporation of nutrient-derived carbons into the tricarboxylic acid cycle [46]. The metabolic phenotypes of neoplastic lymphocytes, and adjacent stroma in DLBCL, indicate an OXPHOS phenotype in neoplastic lymphocytes while stromal cells in DLBCL samples display a glycolytic phenotype [47]. 

**Figure 7:**
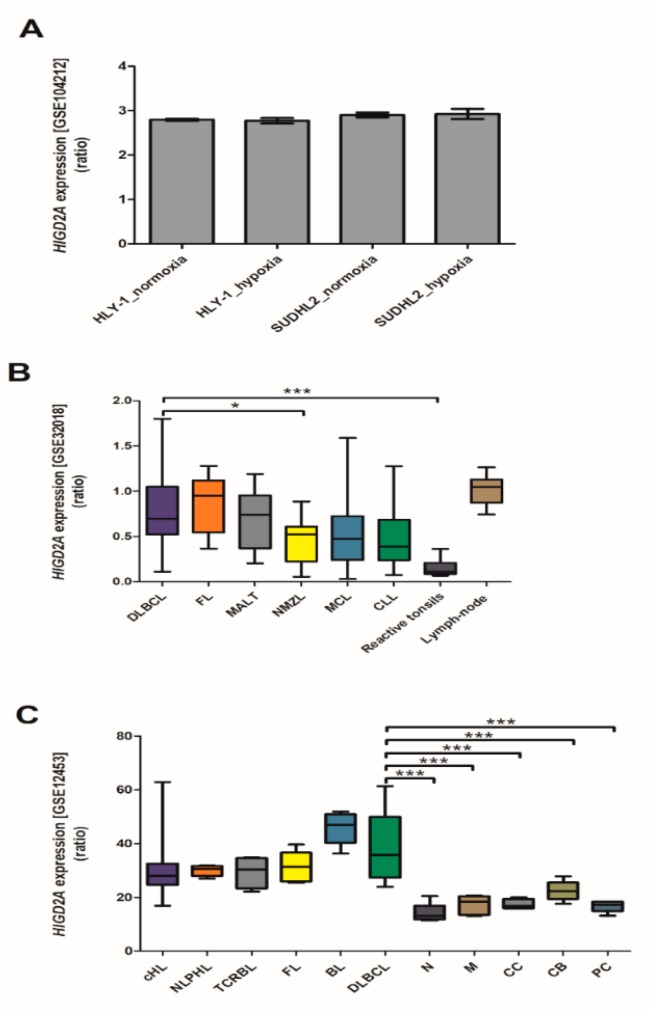
*HIGD2A* expression in DLBCL. (**A**) Dataset GSE104212, Role of hypoxia in Diffuse Large B-cell Lymphoma. Two human lymphoma cell lines, HLY-1 and SUDHL2, were cultured under conditions of hypoxia (*n* = 3) or normoxia (*n* = 3), hypoxia was induced at 1% oxygen in the presence of 5% CO2 for 24 to 48 h [18], and gene expression microarray analysis employed to examine the global gene expression differences under these conditions. (**B**) Dataset GSE32018, Gene-expression profile in a series of non-Hodgkin lymphoma (NHL) patients. FL, Follicular Lymphoma (*n* = 23); MALT, Marginal Zone Lymphoma_MALT type (*n* = 15); NMZL, Nodal Marginal Zone Lymphoma (*n* = 13); DLBCL, Diffuse Large B Cell Lymphoma (*n* = 22); MCL, Mantle Cell Lymphoma (*n* = 24); CLL, Chronic Lymphocytic Leukemia (*n* = 16); reactive tonsils (*n* = 6) and Lymph-node (*n* = 7) were used as controls. (**C**) Dataset GSE12453, Origin and pathogenesis of lymphocyte-predominant Hodgkin lymphoma as revealed by global gene expression analysis. cHL, classical Hodgkin lymphoma (*n* = 12); NLPHL, nodular lymphocyte-predominant Hodgkin lymphoma (*n* = 5); TCRBL, T-cell rich B-cell lymphoma (*n* = 4); FL, Follicular Lymphoma (*n* = 5); BL, Burkitt lymphoma (*n* = 5); DLBCL, Diffuse Large B Cell Lymphoma (*n* = 11); N, Naive B-cells (*n* = 5); M, Memory B-cells (*n* = 5); CC, Centrocytes (*n* = 5); CB, Centroblasts (*n* = 5); PC, Plasma cells (*n* = 5). * *p* < 0.05, *** *p* < 0.001.

Bhalla et al. (2018) [18] studied the role of hypoxia in DLBCL using two human lymphoma cell lines, HLY-1 and SUDHL2, which were cultured under conditions of hypoxia or normoxia. In this study, a gene expression microarray analysis was employed to examine the global gene expression differences under these conditions. In this dataset, we analyzed the *HIGD2A* expression in DLBCL with GEO2R. Neither of the two cell lines displayed differential expression of the *HIGD2A* gene in response to hypoxia (Figure 7A). Bhalla et al. (2018) [18] suggested that the growth of lymphoma cell lines HLY-1 and SUDHL2 was resistant to hypoxic stress. Gómez-Abad et al., (2011) [19] also studied the gene-expression profile in a series of non-Hodgkin lymphoma patients, Follicular Lymphoma (FL), Marginal Zone Lymphoma_Type (MALT), Nodal Marginal Zone Lymphoma (NMZL), Diffuse Large B Cell Lymphoma (DLBCL), Mantle Cell Lymphoma (MCL), Chronic Lymphocytic Leukemia (CLL) and as controls, reactive tonsils, and lymph-node were used. In this dataset, we analyzed the *HIGD2A* expression, and the DLBCL indicated a *HIGD2A* expression significantly higher than the reactive tonsils (Figure 7B). The expression of *HIGD2A* in DLBCL is significantly higher than in NMZL (Figure 7B). Likewise, Brune et al., (2008) [20] studied the origin and pathogenesis of lymphocyte-predominant Hodgkin lymphoma, the analysis of differential gene expression in primary human lymphoma cells of nodular lymphocyte-predominant Hodgkin lymphoma in comparison with primary lymphoma cells of classical Hodgkin lymphoma cells and another B-non-Hodgkin lymphoma, including DLBCL. Furthermore, our dataset analysis reveals a significant higher *HIGD2A* expression in DLBCL concerning all subsets of non- cancerous B lymphocytes isolated from blood or tonsils (naive B-cells, memory B-cells, centrocytes, centroblasts, and plasma cells) (Figure 7C). Lastly, the analysis of the GSE117556 dataset from the retrospective analysis of the whole transcriptome data for 928 DLBCL patients [48] proves no differences of *HIGD2A* expression between the molecular COO subtypes; GCB and ABC.

**Figure 8:**
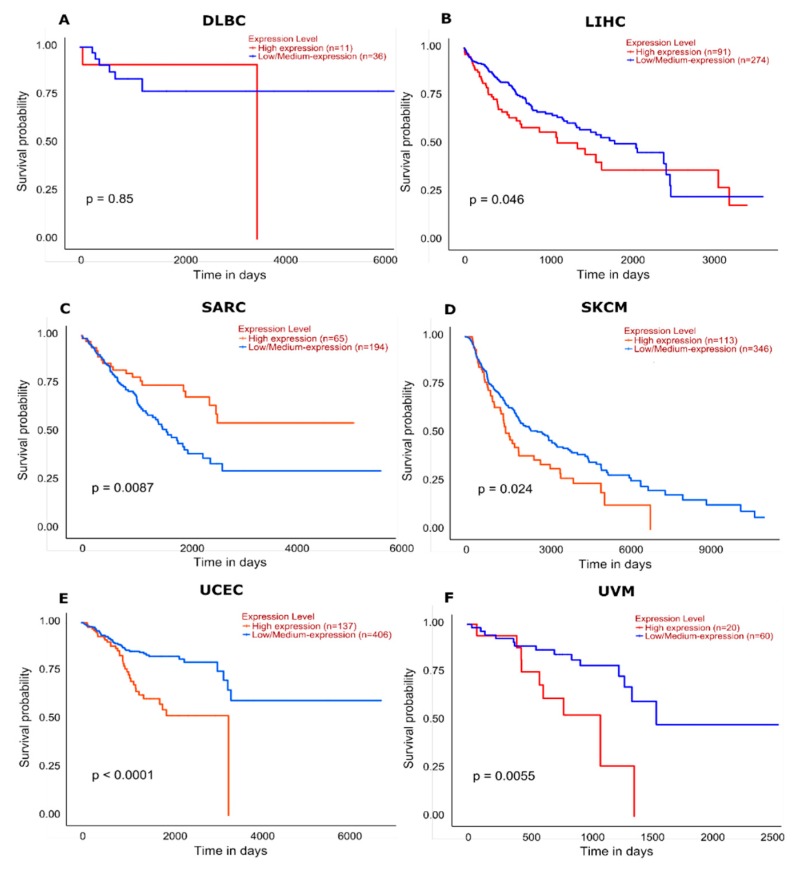
Effect of *HIGD2A* expression on cancer patient survival. The red lines represent a high expression level of *HIGD2A,* and blue lines represent a low/medium expression level of *HIGD2A*. (**A**) DLBCL *p* = 0.85; high expression (*n* = 11), Low/medium expression (*n* = 36). (**B**) LIHC (Liver hepatocellular carcinoma) *p* = 0.046; high expression (*n* = 91), Low/medium expression (*n* = 274). (**C**) SARC (Sarcoma) *p* = 0.0087; high expression (*n* = 65), Low/medium expression (*n* = 194). (**D**) SKCM (Skin cutaneous melanoma) *p* = 0.024; high expression (*n* = 113), Low/medium expression (*n* = 346). (**E**) UCEC (Uterine Corpus Endometrial Carcinoma) *p* ≤ 0.0001; high expression (*n* = 137), Low/medium expression (*n* = 406). (**F**) UVM (Uveal Melanoma) *p* = 0.0055; high expression (*n* = 20), Low/medium expression (*n* = 60).

The effect of *HIGD2A* high expression level on DLBCL patient survival illustrates a downward trend of survival probability in patients (*n* = 11) with high expression in relation with patients (*n* = 36) with low expression, *p* = 0.85 [49] (Figure 8). Other cancers with a high expression of *HIGD2A* present a downward trend survival of patients, being significant for Liver hepatocellular carcinoma (LIHC) *p* = 0.046; Skin cutaneous melanoma (SKCM) *p* = 0.024; Uterine Corpus Endometrial Carcinoma (UCEC) *p* ≤ 0.0001; and Uveal Melanoma (UVM) *p* = 0.0055 (Figure 8). Meanwhile, other cancers with high expression of *HIGD2A* present an upward trend in the survival of patients, being significant for Sarcoma (SARC) *p* = 0.0087 (Figure 8).

### 3.4. Effect of Quercetin on the Expression of Higd2a in Mouse Bone Marrow, Liver and Spleen

**Figure 9:**
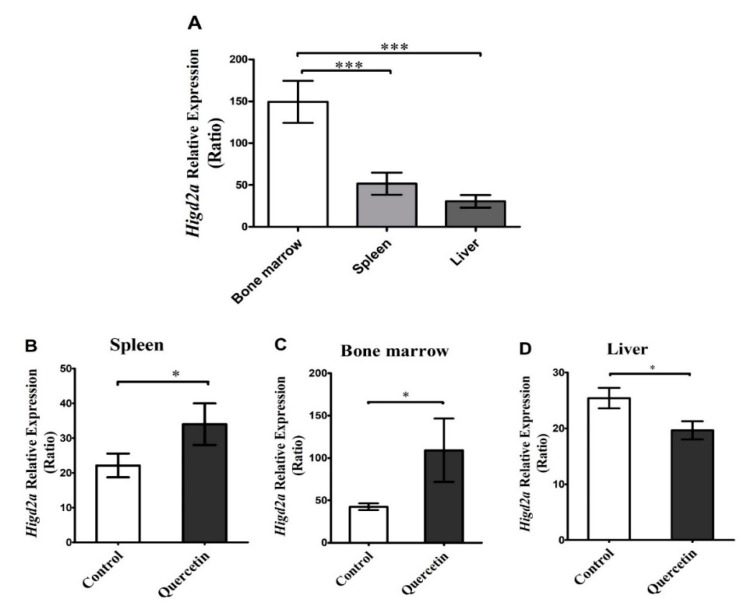
Effect of quercetin on mice *Higd2a* gene expression. Male adult C57BL/6 mice (12 months of age) were administered intraperitoneally daily (i.p.) with 50 mg/Kg quercetin (Cat # Q4951, Sigma-Aldrich) (*n* = 9) or with vehicle (5 % DMSO and PBS) for control animals (*n* = 9), for 15 days. The *Higd2a* gene expression was quantified by RT-qPCR with independent runs of the control spleen, control bone marrow and control liver (**A**). *Higd2a* quantification by Real-Time Quantitative Reverse Transcription Polymerase Chain Reaction (qRT-PCR) with independent runs of the spleen samples of control and quercetin treated; spleen (**B**); bone marrow (**C**) and liver (**D**). Each bar chart represents the mean ± SEM, analyzed by *t*-test (*p* < 0.05), followed by a Mann–Whitney test. * *p* < 0.05, *** *p* < 0.001.

Quercetin is a natural polyphenolic flavonoid, abundant in the human diet, which has several properties: antioxidant, antihypertensive, antifibrotic, antidiabetic, anti-inflammatory, anticancer, and antibacterial [50]. Quercetin has a cancer cell-specific anti-proliferation effect; quercetin has been shown to prevent carcinogenesis in murine models. Quercetin induces anti-proliferation and arrests the G2/M phase in U937 cells; this was associated with a decrease in the E2F1 level [51]. Quercetin induced p21 CDK inhibitor with a related decrease of phosphorylation of pRb, which inhibits the G1/S cell cycle progression by blocking E2F1 [52]. The transcription factor E2F1 is related to the cell cycle. The inhibition of cell proliferation promotes E2F1 binding to the regulatory region of *HIGD2A*, thus setting a role for E2F-1 in the regulation of *HIGD2A* expression [8]. We wonder what would happen with the expression of *Higd2a* in an animal model treated with quercetin, where the cell cycle would present alterations due to quercetin. To this effect, different tissues involved in DLBCL were used: bone marrow, spleen, and liver, from C57BL/6 mice, injected with quercetin (50 mg/kg) and compared with control animals injected with the PBS/DMSO vehicle. The RT-qPCR technique analyzed the expression of the *Higd2a* gene. The relative quantification of the *Higd2a* gene showed tissue specific-differential expression, displaying higher expression in the bone marrow when compared with spleen and liver (Figure 9A). This result may be related to differences in tissues’ proliferation rates. The latter is supported by the findings of Li et al., (2014) [53] who researched the downregulation of survival gene expression of an anti-cancerogenic treatment combined with quercetin. We wondered whether quercetin treatment modulated *Higd2a* expression in relevant tissues for DLBCL. Quercetin significantly increased the expression of *Higd2a* in spleen and bone marrow, while it decreased it in the liver (Figure 9B–D). Finally, the modulation by quercetin of the expression of *Higd2a* in liver, spleen, and bone marrow in adult mice might be related to the effect of quercetin on cellular proliferation.

## 4. Discussion

In this study, we report that the mitochondrial protein HIG2A might have a nuclear localization signal (NLS) and a potential sumoylation motif (Figure 3). The above structural features support the HIG2A nuclear localization, according to our observations made by confocal microscopy and detection of HIG2A in nuclear fractions [8] (Figure 4). HIG2A protein might function as a regulator of respiratory supercomplexes assemblies in response to hypoxia, cellular metabolism, and cell cycle [8]. HIG2A could function as a hypoxia sensor in respiratory supercomplexes to activate signaling pathways of response to hypoxic stress. 

This study focuses on the molecular biosystem analysis of genetic features of the *HIGD2A* gene in cancer biology. We learned that the *HIGD2A* gene is not connected to cancer via mutation. However, DNA methylation and mRNA expression in the *HIGD2A* gene showed significant alterations in diverse cancer (Figure 5). Besides, four miRNAs for the *HIGD2A* gene have been reported as having an influence on cancer development [39,41], summarized in Figure 6 and Table 2. For instance, *HIGD2A* gene showed a significantly higher expression in Diffuse large B-cell lymphoma (DLBCL) (Figure 7). Intriguingly, the *HIGD2A* high expression level on DLBCL patients exhibited a downward trend of survival probability [49] (Figure 8). The correlation of *HIGD2A* high expression and poor patient survival is significant for liver hepatocellular carcinoma; cutaneous skin melanoma; uterine corpus endometrial carcinoma; and uveal melanoma (Figure 8).

In this study, we considerably evaluated the expression of the *Higd2a* gene in healthy bone marrow-liver-spleen tissues of mice after quercetin (50 mg/kg) treatment. The difference in the expression of the *Higd2a* gene in the bone marrow, liver, and spleen may be related to tissues’ proliferation rates (Figure 9). Regardless of liver high metabolic rate, the liver is a quiescent organ (Phase G0 of the cell cycle) with a low rate of cellular proliferation with only 0.0012 to 0.01% of hepatocytes undergoing mitosis [54]. In contrast, the bone marrow is a tissue with a high rate of cellular proliferation of hematopoietic stem cells (HSCs) [55]. Bone marrow presents hypoxic niches [56] that might influence the expression of the *Higd2a* gene. Besides, the generation of red blood cells is stimulated when the blood oxygen levels decay [57].

Recently, a particular type of DLBCL called "bone marrow-liver-spleen" [58,59], which mainly deteriorates those tissues, [60] has been identified. The lymphoid tissues involved in DLBCL display low oxygen levels; bone marrow is hypoxic (pO2 1.3%) with extravascular oxygen tension ranging between pO2 0.6–4.2% [56], spleen also shows a hypoxic environment (pO2 0.5–4.5%) [61]. Meanwhile, the liver presents a higher pO2 of 3–12% [62]. Currently, the importance of hypoxia in this lymphoma has come into play. Hypoxia-Inducible Factor-1 alpha (HIF1α) is stabilized under hypoxic stress in DLBCL cell lines leading to global translational repression that is coupled with a decrease in mitochondrial function [18].

In most growing solid tumors, the vascular aspect is limiting and contains regions that experience hypoxia producing metabolic changes that support energy generation, anabolic processes, and the maintenance of redox potential, thus allowing cancer cells to survive and proliferate in a hostile tumor microenvironment [63,64]. In hypoxia, mitochondria work as an oxygen sensor to regulate cellular energetics, reactive oxygen species, and cell death [65].

In this work, we observed that quercetin modulated the expression of the *Higd2a* gene. In spleen and bone marrow, the expression was increased significantly, while in the liver, it decreased significantly (Figure 9). Modulation of *Higd2a* expression might be related to the effects of quercetin on cellular proliferation in promoting healthy bone marrow mesenchymal stem cell (BMSC) proliferation [66,67]. BMSCs cultured and treated with quercetin (0.1–5 μM and 1–10 μM for the isolation of mouse and rat tissues, respectively) significantly stimulated cells [66,67]. On the other hand, quercetin could have antiproliferative effects [68,69,70,71]. Quercetin at 2 μM shows antiproliferative activity against acute lymphoid leukemia and acute myeloid leukemia [70]. We previously reported that quercetin treatment also affected erythropoiesis. Immature erythroid populations showed a significant increase in the number of cells, while the iron-dependent cell populations of erythropoiesis for heme and hemoglobin biosynthesis significantly decreased in quercetin-treated mice [25]. 

**Figure 10:**
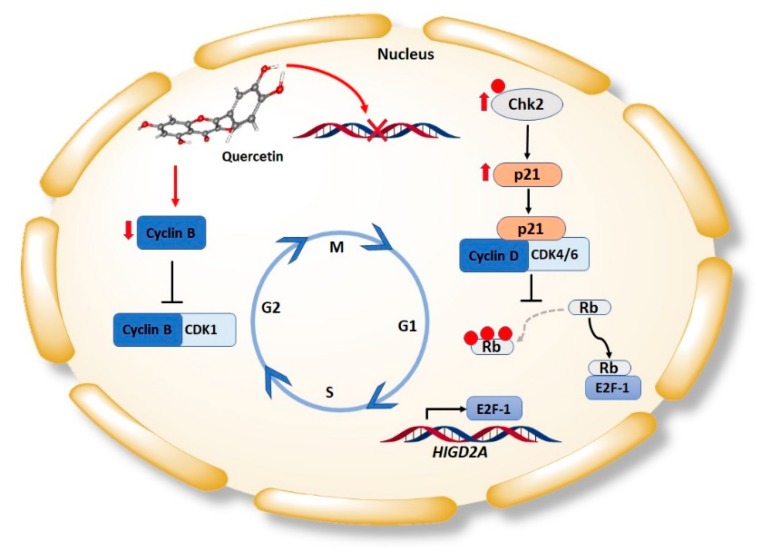
Quercetin can inhibit the progression of the cell cycle in cancer cells. Quercetin induces the arrest of the cell cycle in the G0/G1 phase; Low doses of Quercetin, induces slight damage in the DNA causing the activation Chk2, a primary transcriptional regulator of p21. The p21 protein is a kinase-dependent cyclin inhibitor (CDK), p21 binds to the cyclin/CDK complex in the G1 phase, causing the decrease in the phosphorylation of the retinoblastoma protein (pRb). When pRb is in its hypophosphorylated state, it is bound to the transcription factor E2F1, inhibiting the cell cycle progression in G1 / S, due to the capture of E2F1 by pRb. The transcription factor E2F1 is involved in the regulation of *HIGD2A* gene expression. Quercetin decreases the expression of the cyclin B1 protein by arresting the cell cycle progression in the G2/M phase; Quercetin inhibits the recruitment of the transcription factor NF-Y to the promoter region of the cyclin B1 gene, decreasing its transcriptional expression. Cyclin B1 is an essential component for the function of CDK1 and the progression of the cell cycle in the G2/M phase.

Interestingly, quercetin at 50 μM has an antiproliferative effect on rat splenocytes. These cells have also shown a decrease in cell viability and apoptosis induction [71]. Moreover, human mesenchymal stem cell (MSC), isolated from bone marrow and cultured in the presence of two quercetin concentrations (0.1 and 10 μM), showed that quercetin (10 μM) inhibited cell proliferation of undifferentiated MSC [68]. Furthermore, primary rat hepatic stellate cells (HSCs) and Human LO2 hepatocytes were cultured and treated with quercetin 0.5–120 μM. Quercetin at 20 μM resulted in a significant inhibitory effect of HSC proliferation, and quercetin at concentrations higher than 80 μM significantly inhibited the proliferation of LO2 cells [69]. Besides, quercetin (1–10 μM) exerted inhibition of human breast carcinoma cells proliferation by cell cycle arrest in the G1 phase product of the induction of p21 and a decrease of phosphorylation of the retinoblastoma tumor suppressor protein (Rb) [52] (Figure 10). Remarkably, quercetin at 10 μM did not affect the proliferation of MCF-10A cells, which have the characteristics of normal breast epithelium [52]. All the above indicates that quercetin had selective inhibitory effects on cell proliferation at a specific dose range and suggests that quercetin has a cancer cell-specific anti-proliferation effect.

The transcription factor E2F1 is involved in the regulation of *HIGD2A* gene expression [8] (Figure 10). E2F1 plays a role in energy homeostasis, acting as a metabolic switch from oxidative to glycolytic metabolism under stressful conditions [9,10]. Roscovitine is an inhibitor of CDK that suppresses the proliferation of mammalian cells lines, and roscovitine induced a significant increase in *HIGD2A* gene expression in the human embryonic kidney HEK293 cell line. However, in a mouse myoblast C2C12 cell line, the treatment with Caffeic acid phenethyl ester and Flavopiridol, both antiproliferative agents, decreased *HIGD2A* gene expression [8]. While inhibition of cell proliferation in HEK293 was associated with increased expression of *HIGD2A*, in C2C12 it was associated with *HIGD2A* decreased expression. Therefore, *HIGD2A* expression is not an indicator of cell proliferation.

## 5. Conclusions

DNA methylation and mRNA expression of *HIGD2A* gene present significant alterations in several types of cancer.

Four miRNAs for *HIGD2A* gene show significant gene expression profile related to neoplasms, leukemia, carcinoma, and lymphoma.

*HIGD2A* gene expression is upregulated in DLBCL.

*HIGD2A* gene expression was higher in DLBLC than in Nodal Marginal Zone Lymphoma (NMZL). Although this is not specific for DLBLC, it is a more generalized aspect of cancer cells.

The effect of *HIGD2A* high expression level on DLBCL shows a downward trend of survival probability in patients.

The correlation of *HIGD2A* high expression and poor patient survival is significant for liver hepatocellular carcinoma, skin cutaneous melanoma, uterine corpus endometrial carcinoma, and uveal melanoma.

Quercetin induced the expression of *Higd2a* gene in bone marrow and spleen of healthy mice, while it was reduced in the liver.

It is worth further exploring the role of HIG2A in cancer biology.

## Figures and Tables

**Table 1:** PROCHECK Summary.

	Ramachandran Plot Quality (%)				Goodness Factor		
	Most Favored	Additional Allowed	Generously Allowed	Dis-Allowed	Dihedral	Covalent	Overall
Model-1A	70.3	21.9	7.8	0.0	−0.30	−0.31	−0.29
Model-1B	70.4	23.9	4.2	1.4	−0.27	−0.22	−0.23

**Table 2:** Clinical microRNA (miRNA) and gene expression profile from TCGA (miRNA-Target expression profile).

miRNA. (Accession ID)	Mature miRNA Sequence	miRNA-Target Expression Profile (TCGA)		
		Tumor (*n*)	R (Pearson Correlation)	*p*-Value
hsa-mir-181a-2 (MIRT256742 [miRNA, hsa-miR-181a-5p :: *HIGD2A*, target gene])	39| AACAUUCAACGCUGUCGGUGAGU |61	KICH (25)	0.346	0.05
		PRAD (50)	−0.239	0.05
hsa-mir-181b-1 (MIRT256743 [miRNA, hsa-miR-181b-5p :: *HIGD2A*, target gene])	36| AACAUUCAUUGCUGUCGGUGGGU |58	HNSC (42)	−0.409	3.6 × 10^−3^
		BRCA (84)	−0.258	8.9 × 10^−3^
		KICH (25)	0.374	0.03
		LUSC (38)	−0.276	0.05
		KIRP (32)	0.296	0.05
hsa-mir-181c (MIRT256744 [miRNA, hsa-miR-181c-5p :: *HIGD2A*, target gene])	27| AACAUUCAACCUGUCGGUGAGU |48	BRCA (84)	−0.312	1.9 × 10^−3^
		LIHC (49)	0.283	0.02
		CHOL (9)	0.642	0.03
		LUSC (38)	−0.302	0.03
		KICH (25)	0.346	0.05
hsa-miR-181d (MIRT256746 [miRNA, hsa-miR-181d-5p :: *HIGD2A*, target gene])	36| AACAUUCAUUGUUGUCGGUGGGU |58	BRCA (84)	−0.379	1.9 × 10^−4^
		LUSC (38)	−0.389	7.9 × 10^−3^
		LIHC (49)	0.236	0.05
